# Comparison of Body Weight Support–Assisted Balance and Gait Training With or Without Balance Perturbations in Poststroke Rehabilitation: Multisite Randomized Controlled Trial

**DOI:** 10.2196/80525

**Published:** 2025-12-08

**Authors:** Pete Grevelding, Amanda Meyer, John Corbett, Kaitlyn Rudolf, Emily Meise, Caitlin Boland, Camille Grzelak, Henry Charles Hrdlicka

**Affiliations:** 1 Milne Institute for Healthcare Innovation, Gaylord Hospital, 50 Gaylord Farm Road, PO Box 400, Wallingford, CT, 06492, United States, 1 203-284-2864; 2 Inpatient Occupational Therapy, Hartford Hospital, Hartford, CT, United States; 3 Inpatient Physical Therapy, Gaylord Hospital, Wallingford, CT, United States; 4 Inpatient Occupational Therapy, Gaylord Hospital, Wallingford, CT, United States; 5 Inpatient Physical Therapy, MedStar National Rehabilitation Hospital, Washington, DC, United States

**Keywords:** stroke rehabilitation, postural balance, gait, ambulation, balance perturbation, postural perturbation, body weight support system, physical therapy, long-term acute care hospital, acute rehabilitation

## Abstract

**Background:**

Impaired balance regulation after stroke puts patients and therapists at a heightened risk of injury during rehabilitation. Body weight support systems (BWSS) allow patients to safely conduct gait and balance training while minimizing risk and the fear of falling. Integrating perturbation-based balance training (PBT) modules with a BWSS may lead to further improvements.

**Objective:**

This study aimed to evaluate the impact of a ceiling-track-mounted BWSS plus an integrated PBT (BWSS-P) module on the rehabilitation of stroke-related gait and balance impairments.

**Methods:**

A multisite randomized active-comparator controlled trial was conducted. Inpatients with Berg Balance Scale (BBS) scores ≥21/56 were recruited from 4 sites. Participants completed 2-to-6 BWSS or BWSS-P study sessions. Both groups conducted the same balance and gait regimen, with the BWSS-P group also receiving 8 balance perturbations per session. BBS, Activities-Specific Balance Confidence, and 10 Meter Walk Test assessments were collected.

**Results:**

A total of 126 patients were approached with 99 yielding sufficient data for analysis. With a corrected *α* level of .0054, both groups showed significant in-group changes over time for all outcomes evaluated (*P*≤.001). However, the primary outcome measure, the BBS, did not show evidence of a difference between BWSS and BWSS-P groups over time (*F*_1,97_=1.57; *P*=0.21) via linear mixed effects modeling with type III Wald *F* test with Kenward-Roger degrees of freedom. No significant between-group differences were noted for either secondary outcome measure, the Activities-Specific Balance Confidence scale (*F*_1,94.99_=0.36; *P*=.55), or 10 Meter Walk Test (*F*_1,97_=4.15; *P*=.04).

**Conclusions:**

Although significant between-group differences were not observed, participants in both groups demonstrated similar improvements from before to after assessment. This novel PBT technology shows promise as an option for gait and balance recovery training, especially for individuals with impaired balance control or fear of falling. The BWSS-P intervention contributed positively to rehabilitation outcome overall and should be considered as a viable treatment modality when clinically appropriate.

## Introduction

Each year, more than 795,000 people experience a stroke [1]. Strokes are devastating neurological events that can lead to cognitive and physical deficits, such as the inability to ambulate, impaired balance regulation, and loss of coordination [2]. Following a stroke, many patients are admitted to rehabilitation facilities to address these deficits and maximize their independence before returning to the home setting [3]. Gait and balance dysfunction are common secondary impairments to stroke, usually requiring specific neurological rehabilitative interventions to regain functional independence [4,5].

As a result of these physical deficits, patients are at a higher risk of experiencing a fall following a stroke [6]. Moreover, fear of falling has also been shown to influence balance and gait control in older adults, with over 90% of survivors of stroke reporting that the fear of falling negatively impacts their performance of daily living activities [7]. These psychological factors are strong predictors of falling compared to some physical factors or the presence of pathology [8,9]. It is reasonable then to suspect that any fall that occurs during rehabilitation would either reinforce or create a psychological fear of falling that would impede patient recovery [9].

Unfortunately, falls during and outside of rehabilitation can prolong patients’ inpatient length of stay, increasing the economic and financial strain on them and their families when the patient is unable to work [10]. Any falls, especially injurious falls, also increase the financial burden of an institution [11]. Focusing on balance and gait training early in patients’ rehabilitation can improve their overall fall risk, reducing the need for other resources such as safety sitters and further improving the financial burden of the institution. One strategy for improving balance and gait training safely is to incorporate new and existing robotic technologies into neurological rehabilitation, including body-weight support systems (BWSS) [12].

BWSS are stationary or track-mounted suspended harnesses that support a predetermined portion of a patient’s body weight. BWSS permits those with significant weakness and poor coordination to ambulate and perform more intensive therapy sessions sooner in their recovery with minimal risk of injurious falls [13]. Current evidence supports the safety and effectiveness of BWSS during rehabilitation training, showing greater improvements in patient outcomes in patients treated with BWSS versus without [14,15].

Perturbation-based balance training (PBT), the practice of purposefully unbalancing patients to rehabilitate their postural control, can also be used to improve patients’ balance control following stroke or other age and disease-related balance impairments [16-21]. Where other training modalities can only provide nonreactive balance training, PBT can be used to improve both reactive and anticipatory control to unexpected and anticipated stimuli [22]. Conventionally, PBT is conducted using modified treadmills, unstable surfaces, or external force directly provided by a treating therapist [16-21]. These strategies present a risk to patients and therapists alike. Incorporating a stationary BWSS over a modified treadmill or unstable surface can decrease this risk; however, ambulation over a treadmill does not represent functional ambulation in the patients’ home environment, and some patients struggle to use a treadmill. Unfortunately, there is little consensus about the optimal training regimen for PBT [23,24].

To reduce risk and replicate patients’ functional ambulation, new devices have been developed that integrate PBT with an overhead track-mounted BWSS [12,25,26]. Using this “fall-free” environment, therapists can deliver PBT safely and consistently earlier in patients’ plan of care. Previously, we conducted a pilot study of such a device [13].

In that study, we demonstrated that a standardized balance and gait training regimen using BWSS or BWSS plus an integrated PBT module (BWSS-P) was safe and did not negatively impact patient recovery [13]. The preliminary efficacy data from this pilot study demonstrated similar improvements in BWSS and BWSS-P groups from baseline to postintervention, but as a pilot study, it was underpowered to determine if there was a significant impact on patient outcomes between groups.

The goal of this study was to build upon this pilot study and conduct a definitively powered randomized controlled trial. We hypothesized that BWSS-P would demonstrate greater outcomes than BWSS-supported training alone.

## Methods

### Ethical Considerations

A multisite randomized active-comparator controlled clinical trial was conducted. The study was submitted, reviewed, and approved by each site’s institutional review board for either local review or to complete a reliance agreement with the Gaylord Hospital institutional review board [27]. Enrollment began on December 15, 2021, and ended on November 6, 2023. Once approved, the study procedures followed were in accordance with the ethical standards of the responsible committee on human experimentation (institutional and national) and with the Declaration of Helsinki. This included maintaining the privacy and confidentiality of research participants’ data and identity. Written informed consent was delivered and collected from each participant before study activities were conducted. Participants were not compensated for study participation. Before the study was initiated, the study was prospectively registered with ClinicalTrials.gov (NCT05110300) on October 26, 2021. For reporting, the CONSORT (Consolidated Standards of Reporting Trials) framework was used (Checklist 1).

### Power Analysis and Interim Analysis

Referencing the early efficacy data collected in the pilot study [13], a Cohen *d* effect size of 0.39 was calculated for the observed Berg Balance Scale (BBS) change using a 2-sample, 2-tailed *t* test. Assuming a similar effect size, a 2-tailed power analysis (*α*=.05) and sample size estimation for 80% power were conducted. Using G*Power, an estimated sample size of 214 participants was needed for this effect size to achieve 80% power. During study development, an interim analysis was prospectively planned once 100 participants successfully completed the study activities. Given the small effect size noted in preliminary efficacy data, the goal of this interim analysis was to evaluate the study progression and to determine where there was any clear evidence of a clinically important difference between intervention groups that was or was not trending towards statistical significance.

### Study Locations and Study Criteria

Participants were recruited from 1 of the 4 participating inpatient rehabilitation centers: Gaylord Specialty Healthcare, Wallingford, CT (site 1); MedStar National Rehabilitation Hospital, Washington, DC (site 2); Spaulding Rehabilitation Hospital Cape Cod, Sandwich, MA (site 3); and St. Luke’s Rehabilitation Hospital, Spokane, WA (site 4). Site 1 was the primary coordinating site for study activities.

To be eligible, participants had to be admitted for inpatient rehabilitation following either an ischemic or hemorrhagic stroke of any laterality or location. Patients were evaluated by physical therapy within the first 72 hours of admission at each recruitment site, at which point an initial BBS was obtained as appropriate. To be eligible, patients had to have a BBS score of ≥21/56. The BBS was chosen as the primary eligibility criterion as it was the standard functional balance measure used across all sites.

Patients were ineligible to participate if they did not meet any one of these criteria or presented with one or more of the exclusion criteria shown in Textbox 1. To increase recruitment, the progression of patients with a BBS of <21 at admission was tracked; if at any point those patients later progressed to a BBS of ≥21/56, they were rescreened for study inclusion. If the patient met the study criteria, did not meet the exclusion criteria, and had a set discharge date of 10 days or longer from the time of recruitment, they were consented into the study.

Textbox 1. **Exclusion criteria.**

- Cognitive deficits that would disrupt the ability to provide informed consent
- Active enteric infection control precautions
- Ongoing orthostasis
- Uncontrolled hyper- or hypotension
- Active seizures
- Spinal stabilization with the use of a halo-brace or halo-gravity traction
- Unstable skin structures (ie, skin grafts)
- Chest tubes
- Unstable rib or lower extremity fractures
- Severe osteoporosis
- Subjects where pressure around the abdomen, thighs, groin, or shoulders is contraindicated
- New limb amputations
- Vestibular disorders that may impact balance
- Premorbid conditions that may impact balance
- Patients requiring more than 50% high flow oxygen as consistent with inpatient therapy guidelines.
- Subjects who weigh more than 450 pounds, per the structural limitations of the ZeroG system.
- Anyone belonging to a vulnerable population, including individuals aged <18 years and who are or might be pregnant.

### Functional Outcome Instruments

The primary end point for this study was the BBS [28], with the Activities-Specific Balance Confidence (ABC) scale [29], and the self-selected 10 Meter Walk Test (10MWT) as secondary outcomes [30]. The BBS is an objective clinician-reported balance measure with a scale of 0 to 56 (0 being the lowest function and 56 being the highest function), the ABC scale is a subjective patient-reported balance confidence measure with a scale of 0% to 100% (0% being the least confident and 100% being the most confident), and the self-selected 10MWT is used to evaluate gait speed in meters per second (m s^–1^), which has been shown to correlate to balance and fall risk [31].

The BBS evaluation that was collected within 72 hours of admission was used as the baseline assessment (ie, before assessment) during data analysis. If eligible and willing to participate, participants were consented and enrolled into the study within 48 hours of admission.

After consent was collected, and before the study-related therapy sessions began, the ABC scale and 10MWT were administered by site investigators. Within 48 hours of the last study session or prior to discharge, whichever occurred first, a post-intervention assessment (ie, after assessment) was collected for all measures.

### BWSS Equipment

For this study, the ceiling-track-mounted, Food and Drug Administration–listed ZeroG Gait and Balance BWSS and the ZeroG Training Responses in Postural Rehabilitation balance perturbation module (Aretech LLC) were used to complete this study. All study locations already had the ZeroG BWSS with Training Responses in Postural Rehabilitation module installed prior to study initiation. The study sites were not sponsored or supported by the manufacturer to conduct this study.

### Standard Care Protocol and PBT Interventions

To minimize disruptions to participants’ standard therapy regimens, and to maximize the generalizability of the study protocol in real-world settings, the pragmatic decision was made to integrate study-related sessions directly into the participants’ standard care routines. As part of their normal care, participants received a minimum of 3 hours of multidisciplinary inpatient physical, occupational, or speech rehabilitation appropriate for their condition, and as was prescribed by their attending physician. This standard of care did not include additional PBT.

As appropriate, participants’ standard of care therapy regimen included, but was not limited to, functional mobility training targeting specific activities of daily living and instrumental activities of daily living needs, endurance training, balance retraining, coordination retraining, strengthening, high-intensity gait training, range of motion training, patient education with durable medical equipment, and family training prior to discharge. Referrals and treatment by other specialties (ie, therapeutic recreation, dietary, and neuropsychology) continued as normal.

Study-related therapy sessions were conducted individually. Each session lasted a minimum of 15 minutes and a maximum of 30 minutes. Data were collected from all participants who completed at least 2, and up to 6, study-related therapy sessions. Data from a range of completed sessions were purposefully collected to accommodate “real-world” short or unexpected changes to discharge timelines due to insurance authorization or other disturbances. This allowed us to examine the effect of sessions completed on outcome measures recorded. Based on the average length of stays of the study sites, once consented and enrolled in the study, participants received an average of 3 study sessions per week, approximately one every other day, until discharge.

During the BWSS only control group sessions, participants conducted a series of standard balance exercises, including: marching, side-stepping, retroambulation, step taps, and step-ups. Standard gait exercises were also completed, including: overground ambulation, going up and down stairs, and sit-to-stand transitions. For each participant, only 4.54 kg (ie, 10 pounds) of body weight was unloaded as this is the minimum requirement to activate the BWSS. As this is an active clinical comparison, clinical parameters and progression, other than those described earlier, were determined by the treating clinical investigator (ie, use of assistive devices and bracing) to ensure the participant’s safety, success, and appropriate challenge.

The BWSS-P group completed the same balance and gait exercises as the control group; they differed only in the inclusion of 8 total balance perturbations, 2 in each cardinal direction (lateral left, lateral right, anterior, and posterior). Perturbations occurred while static (lateral, anterior, and posterior) and dynamically while ambulating (anterior and posterior). Eight perturbations were selected based on the findings of the previous pilot study [13] and to minimize fatigue effects. In that study, we found that 1 perturbation every 2 to 4 minutes during the 15-to-30 minute study-related session was enough to challenge participants without overtaxing them and allow for a wider range of participants with varying tolerances, while also allowing them to conduct other elements of their physical therapy regimen.

Perturbation intensity was determined by participants’ tolerance and ability to recover from perturbations at baseline. At the start of the study, a baseline BWSS-P perturbation level was individually set for each participant by incrementally increasing the level to balance loss or near loss without recovery. The perturbation level then was decreased by 1 level for treatment. All 8 perturbations were set to this level until it was clinically appropriate to progress to maintain a sufficient challenge to the patient. The intensity of the perturbation ranged from level 1 (minimum) to level 10 (maximum), as set by the manufacturer. Note, while both groups had access to assistive devices during ambulation as appropriate to ensure participant safety, their use was minimized and was not used at all during balance perturbations. Furthermore, the BWSS group did not receive any balance perturbations, including manual perturbations delivered by the treating clinician.

Treating clinicians were licensed physical therapists who were all trained on best practices in using the specific BWSS and perturbation module. To ensure consistency, an initial virtual training session was led by site 1 to review best practices for delivering all study outcome measures. A virtual training session was agreed upon as COVID-19-related travel and visitation restrictions limited the lead site’s ability to conduct in-person site initiation visits. Following this, site 1 led a monthly steering committee meeting that consisted of each study site principal investigator, representatives of the treating clinical teams, and the study’s acting data and safety monitor. The goal of this meeting was to review if any safety events or harm had occurred, discuss protocol adherence, answer any clinical or technical questions, discuss recruitment, and address any other items as needed.

### Participant Recruitment, Enrollment, and Randomization

Each study site was responsible for screening, recruiting, and consenting qualifying participants from their individual locations. Once consented, participants were randomly assigned to either the BWSS standard intervention (ie, control) group or the BWSS-P intervention (ie, experimental) group according to a pregenerated assignment sheet provided by the coordinating site. Each sheet was generated using block randomization using the random list generator tool found at Random.org. A block size of 4, 2 BWSS, and 2 BWSS-P assignments per block were used. Depending on anticipated recruitment numbers, 12-to-20 assignments were generated for each site at a time to allow for balancing as needed.

Given the nature of the intervention (ie, whether or not a baseline perturbation level was needed), clinical staff and participants were unable to be blinded to the randomly assigned group assignments.

### Data Analysis

Data were analyzed using GraphPad Prism (version 10; GraphPad Software) and RStudio (version 4.2.2, Posit Software) using the *car*, *lme4*, and *emmeans* packages [32-34].

Descriptive data were reported as mean (SD). If hypothesis testing was done, data were reported with a *P* value, and significance was set at *α*=.0054 using the O’Brien-Fleming correction for interim analysis [35]. The primary outcome measure was the BBS. Secondary outcome measures included the ABC and 10MWT. Data were determined to be missing completely at random and were dealt with via complete case analysis for the total outcome measure change [36].

Pairwise categorical data were analyzed using chi-square testing. Unpaired 2-tailed *t* tests or Mann-Whitney *U* tests were used as necessary to evaluate pairwise between-group differences in continuous outcome variables. 2-way ANOVA or mixed-effects analysis was used to evaluate pairwise pre- and postoutcome measure data.

Intervention effect on outcome measure data was analyzed using a linear mixed effects model. In the models, outcome measures were the dependent variable, with fixed effects for timepoint, group assignment, sessions completed, study location, and timepoint x group assignment interaction term. Participant was included as a random effect. Type III Wald *F* test with Kenward-Roger degrees of freedom was used to evaluate the significance of all the fixed effects. Model assumptions were assessed through visual inspection of residual plots and model diagnostics.

## Results

### Participant Characteristics

**Figure 1.**
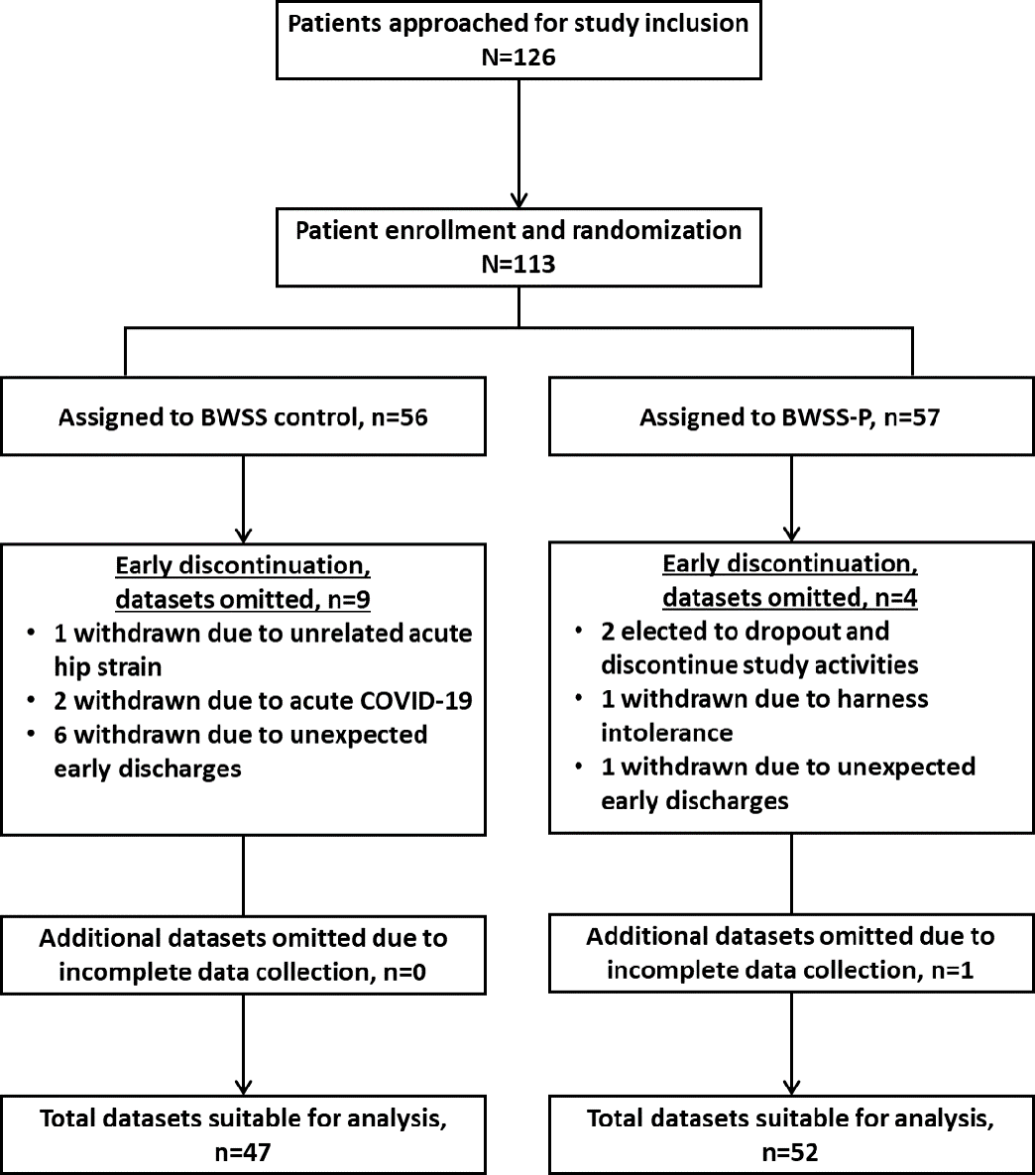
CONSORT (Consolidated Standards of Reporting Trials) diagram. The diagram outlines how many eligible patients were approached, recruited, and completed the study. It also indicates the number of datasets excluded from analysis and the rationale for doing so. BWSS: body weight support system; BWSS-P: body weight support system plus an integrated perturbation-based balance training module.

The study’s initial target was a final sample size of 214 participants. However, based on the results of the planned interim analysis following the recruitment and completion of 100 participants, the study team made the decision to end study recruitment early and proceed with full data analysis using an appropriately adjusted α. In total, 126 participants were approached for study inclusion, 113 were consented, enrolled, and randomized, with 99 participants yielding sufficient data for analysis (Figure 1). No major or serious adverse events were noted during the study period.

Of the 99 participants with complete data, 47 were assigned to the BWSS only control group, and 52 were assigned to the BWSS-P group. No trends related to sex, age, or any other demographic were observed between groups (Table 1). Participants began study activities a mean of 16.00 (SD 8.01) days following their stroke and a mean of 1.99 (SD 1.99) days following collection of the BBS preintervention assessment.

**Table 1.** Patient demographics (N=114).

	BWSS^a^ (n=47)	BWSS-P^b^ (n=52)	*P* value
Participants, n (%)^c^			.730^d^
Site 1	21 (45)	20 (38)	
Site 2	13 (28)	16 (31)
Site 3	8 (17)	7 (13)
Site 4	5 (11)	9 (17)
Age (y), mean (SD)	62.36 (14.13)	65.63 (12.55)	.229^e^
LOS (d)^f^, mean (SD)	18.34 (8.45)	16.42 (7.71)	.171^g^
Sex, n (%)			.887^d^
Male	31 (66)	35 (67)	
Female	16 (34)	17 (33)
Race, n (%)			.700^d^
African American	12 (26)	16 (31)
Caucasian	33 (70)	35 (67)
NR^h^	2 (4)	1 (2)
Total sessions completed, n (%)			.476^d^
2 sessions	5 (11)	3 (6)	
3 sessions	8 (17)	11 (21)
4 sessions	11 (23)	16 (31)
5 sessions	10 (21)	14 (27)
6 sessions	13 (28)	8 (15)
Sessions completed, mean (SD)	4.38 (1.34)	4.25 (1.14)	.595^e^

a BWSS: body weight support system.

b BWSS-P: BWSS plus an integrated perturbation-based balance training module.

c Due to rounding, percentages may not total 100%.

d Chi-square test.

e Unpaired *t* test.

f LOS: length of stay.

g Mann-Whitney *U *test

h NR: not reported.

### Participant Outcome Measures

**Figure 2.**
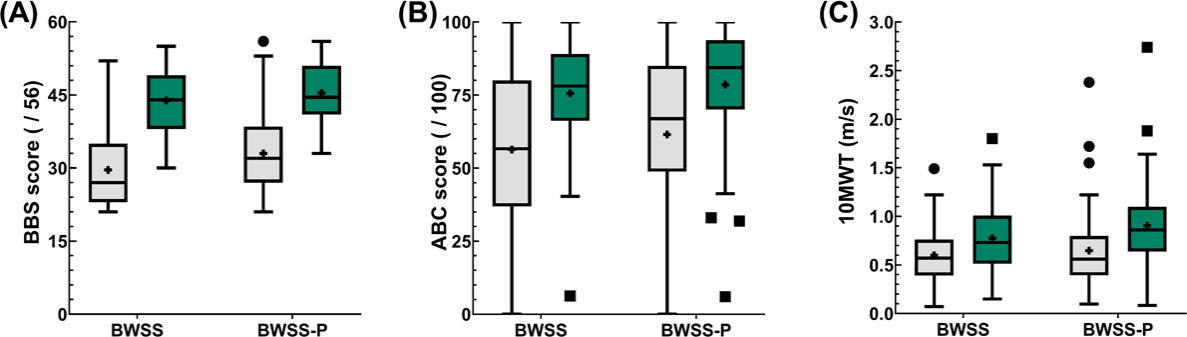
Participant outcomes. Primary outcome measures were collected for both groups at baseline and after completing the study protocol, including the (A) Berg Balance Scale (BBS); (B) Activities-Specific Balance Confidence (ABC) Scale; and (C) self-selected 10 Meter Walk Test (10MWT). Data are presented as box-and-whisker plots with the box representing the 25th to 75th quartiles, the midline representing the median, the (+) symbol representing the mean, the whiskers denoting the minimum and maximum of the dataset excluding any outliers, and the open circles representing individual outliers. The gray or lighter color represents the preintervention data, while the green or darker shading represents the postintervention data. BWSS: body weight support system; BWSS-P: BWSS plus an integrated perturbation-based balance training module.

The outcome measures collected included baseline and post-intervention assessments of BBS, ABC, and 10MWT (Figure 2). The effect of the BWSS-P intervention was analyzed using linear mixed effects models with a type III Wald *F* test with Kenward-Roger degrees of freedom for all outcome measures collected. With a corrected *α* level of .0054, the intervention did not show any significant impact on our main outcome measure, the BBS. This was shown by the *time x assignment interactions* (*F*_1,97_=1.57; *P*=.21). Similarly, no significant intervention effects were observed in either the ABC (*F*_1,94.99_=0.36; *P*=.55) or 10MWT (*F*_1,97_=4.15; *P*=.04; Table 2).

**Table 2.** Model effects.

Parameter	Fixed effects^a^ of BBS^b^^,^^c^			Fixed effects of ABC^d^^,^^e^			Fixed effects of 10MWT^f^^,^^g^		
	*F* test (*df*)		*P* value	*F* test (*df*)		*P* value	*F* test (*df*)		*P* value
Time x assignment	1.57 (1,97)		.21	0.36 (1,94.99)		.55	4.15 (1,97)		.04
Time	181.01 (1,97)		<.001^h^	42.89 (1,95.30)		<.001^h^	23.71 (1,97)		<.001
Assignment	5.92 (1,148.14)		.02	1.09 (1,132.74)		.30	0.57 (1,110.82)		.45
Sessions completed	6.23 (1,93)		.01	3.29 (1,93.25)		.07	0.57 (1,93)		.45
Location	3.12 (3,93)		.03	0.02 (3,92.83)		.99	3.44 (3,93)		.02
Random effects^a^	Variance		SD	Variance		SD	Variance		SD
Participant	26.80		5.18	342.3		18.50	0.12		0.35
Residual	26.30		5.13	198.6		14.09	0.03		0.16

a Results from linear mixed effects models. Fixed effects were tested using a type III Wald *F *test with Kenward-Roger degrees of freedom. Random effects are shown as variance with SD.

b BBS: Berg Balance Scale.

c In BBS, random effects of participant: variance 26.80 (SD 5.28) and residual: variance 26.30 (SD 5.13).

d ABC: Activities-Specific Balance Confidence.

e In ABC, random effects of participant: variance 342.3 (SD 18.50) and residual: variance 198.6 (SD 14.09).

f 10MWT: 10 Meter Walk Test.

g In 10MWT, random effects of participant: variance 0.12 (SD 0.35) and residual: variance 0.03 (SD 0.16).

h Significance was set at *α*=.0054 using the O’Brien-Fleming correction for interim analysis.

Time effects were significant across all outcome measures. BBS scores showed significant improvement from admission to discharge across both groups (*F*_1,97_=181.01; *P*<.001), along with ABC (*F*_1,95.29_=42.91; *P*<.001) and 10MWT (*F*_1,97_=23.71; *P*<.001).

Assignment effects were nonsignificant across all outcome measures. BBS showed the most suggestive evidence of differences between groups (*F*_1148.14_=5.55; *P*=.02), with ABC (*F*_1132.74_=1.09; *P*=.30) and 10MWT (*F*_1110.82_=0.57; *P*=.45) outcomes showing less evidence of a difference.

The number of sessions completed (ie, 2-to-6) did not have any evidence of a significant effect on the outcome measures. BBS showed the most difference in regards to the number of sessions completed (*F*_1,93_=6.23; *P*=.01), with ABC (*F*_1,93.25_=3.29; *P*=.07) and 10MWT (*F*_1,93_=0.57; *P*=.45) showing smaller differences.

Location had similarly suggestive, but inconclusive, evidence of an effect on outcome measures. BBS (*F*_3,93_=3.12; *P*=.03) and 10MWT (*F*_3,93_=3.03; *P*=.03) had the most evidence of a location effect, while ABC did not (*F*_3,92.83_=0.01; *P*=.99).

Model estimated marginal means (SE) for the BBS showed that at admission, both the BWSS (30.02, SE 1.15) and BWSS-P (33.44, SE 1.13) groups had similar scores. This finding was also true at discharge, with BWSS (43.45, SE 1.15) and BWSS-P (45.42, SE 1.13) ending at a similar level. Similar patterns were noted in the model estimated means of the secondary outcome measures, as well as the observed unadjusted means for all outcomes (Table 3).

**Table 3.** Outcome measure pre- and post-assessment scores.

Outcome measure and type		Before assessment, mean (SD), n	After assessment, mean (SD), n	*P* value
BBS^a^ (0-56)				
BWSS^b^				
Observed		29.62 (8.58), 47	43.85 (6.62), 47	<.001^c^
Estimated**^d^**		30.02 (1.15)	43.35 (1.15)	<.001**^e^**
BWSS-P^f^				
Observed		33.00 (8.62), 52	45.40 (6.32), 52	<.001
Estimated		33.44 (1.13)	45.42 (1.13)	<.001
ABC^g^ (/100)				
BWSS				
Observed		56.35 (25.26), 47	75.58 (18.12), 45	<.001
Estimated		58.18 (3.74)	76.04 (3.81)	<.001
BWSS-P				
Observed		61.53 (27.50), 51	78.54 (20.05), 52	<.001
Estimated		62.96 (3.69)	78.77 (3.68)	<.001
10MWT^h^ (m s^–1^)				
BWSS				
Observed		0.60 (0.33), 47	0.77 (0.36), 46	<.001
Estimated		0.58 (0.06)	0.73 (0.06)	<.001
BWSS-P				
Observed		0.65 (0.42), 52	0.90 (0.45), 52	<.001
Estimated		0.66 (0.06)	0.91 (0.06)	<.001

a BBS: Berg Balance Scale.

b BWSS: Body weight support system.

c *P* values from two-way ANOVA and mixed-effects analysis as applicable.

d Values shown are estimated marginal means (SE) from the mixed effects model. These values are predictions estimated from the fitted model. Since they are estimates, there are no sample sizes associated with these reported values.

e *P* values from type III Wald *F *test for main effect of time in linear mixed effects model

f BWSS-P: BWSS plus an integrated perturbation-based balance training module.

g ABC: Activities-Specific Balance Confidence Scale

h 10MWT: 10 Meter Walk Test.

## Discussion

### Principal Findings and Alignment With Literature

The goal of this study was to compare the effects of a BWSS-integrated PBT training module on poststroke balance and gait rehabilitation as compared to BWSS training alone. Specifically, participants completed a regimented neurological rehabilitation program that included 2-to-6 BWSS sessions with or without 8 balance perturbations per session. Participants in both groups demonstrated similar within-group improvements with no significant between-group differences in BBS, ABC, or 10MWT. Furthermore, there was suggestive, but inconclusive, evidence of minimal site-specific effects. While these observations are nonsignificant and confirm the null hypothesis that the BWSS-P intervention is not different than the BWSS only control, these data still provide us with meaningful observations that support past publications.

First, similar to past observations [37], the number of sessions that participants completed greatly varied due to several extenuating and pragmatic factors, including insurance continuing stay authorization [37]. While not ideal, these data and the protocol are reflective of what are feasible and realistic best practices in these inpatient settings in the current environment.

Our findings are also in alignment with other past studies that tested the effect of traditional PBT intervention in the poststroke population [17,19,20,38-44]. Many of these studies used the same outcome measures that were used in this study. Of these, 6 studies used the BBS to evaluate balance [19,20,39,40,42,43], 2 studies used the 10MWT to assess gait speed [17,41], and 2 studies used the ABC to measure participants’ balance confidence [17,41]. Similar to our findings, little to no between-group differences were observed for each of these outcome measures.

As noted by Brown et al [44], these past studies all varied in training duration, frequency, and interventions used. Study sessions ranged from 1 to 24 sessions, and the number and type of perturbations also varied and included treadmill delivery, therapist-applied perturbations, waist pull perturbations, and slip training, in addition to other commercially available systems. Few studies, however, have examined the impact of overhead gait robotics that facilitate balance perturbation. These studies and others report encouraging results; however, the impact of PBT on stroke balance rehabilitation remains inconclusive [20,22,44].

In addition to unifying study interventions, Mcrum et al [22] have also indicated the need for further dosing studies to examine optimal dosing within sessions or within training programs in addition to examining the effect of bidirectional and multidirectional perturbation training to provide recommendations for clinicians. Following this study, 1 observation that we made is that the level of perturbation experienced by the end user can be defined in many ways, including the magnitude and duration of the applied force that disrupts the user’s balance. This can be controlled by factors like the force applied, the direction of the force (eg, forward, backward, and lateral), and the timing of the perturbation during the gait cycle. In addition, the level of body weight support provided by a harness can also influence the perceived perturbation level, as less support means greater impact from the applied force, and more support may result in the patient being able to complete greater repetitions. These factors all need to be taken into account when planning future dosing studies of this technique.

### Limitations and Lessons Learned

As this was the first multisite randomized-controlled trial to evaluate this novel BWSS-integrated PBT technology, we can identify several limitations that must be considered when interpreting the data and planning future studies.

First, since the study was conducted in the inpatient rehabilitation setting, the study activities were only a small part of the participants’ overall treatment strategy. Several factors, including therapy outside the study and natural recovery progression, likely contributed to improvements in participants’ status and function. Despite attempting to unify the delivery of the study intervention, treatment strategies outside of study activities may have been variable across study sites. For example, in seeking possible explanations of the suggestive location effect, it was found that site 2 had implemented an institution-wide high-intensity gait training program as part of their site’s standard of care, which was not used by the other study sites during the study.

Second, BBS score changes may have been limited due to ceiling effects. To address this, an upper BBS score should be considered as part of the study criteria in all future studies on this topic. For example, a BBS score of 45/56 has been suggested to describe patients with normal functional ability [28]. Similarly, incorporating a dynamic gait assessment that more closely resembles functional movement patterns and reactive balance-specific outcome measures may be beneficial to understanding the full implications of this new BWSS-P module [45,46].

Third, several participants (8/99, 8%), constrained by short lengths of stay, only completed 2 sessions, the minimum number to be included in the study. This limited dose may not have been enough to elicit a clinically significant change in balance and balance confidence as may have been hypothesized. Short lengths of stay were due to several factors, including industry trends of decreasing lengths of stay in the cerebrovascular accident (ie, cerebrovascular accident, stroke, and brain injury) population; inclusion of participants with BBS scores of 45 or greater; and the impact of COVID-19. Specific to COVID-19, many patients opted to discharge home as soon as they could safely navigate the home environment with assistance, meaning some patients did not remain long enough to receive more than 2 study sessions. In addition, many patients who were eligible for the study could not participate due to a positive COVID-19 diagnosis and droplet precautions, while others were withdrawn and their data omitted from analysis due to breakthrough infections requiring isolation.

Fourth, the number of balance perturbations provided to the BWSS-P group was strictly kept to 2 balance perturbations in each cardinal direction per session (ie, 8 total). This was maintained even if the first or second perturbation in a particular direction did not elicit a strong balance response. Further, a target of 6 study sessions was based on the preliminary efficacy data collected during the pilot study; this may not have been as optimal as previously thought [13]. In future studies, it will be essential to expand upon this work and complete a dedicated dosing study to determine the optimal number of BWSS-P sessions and number of balance perturbations per session [22].

Fifth, the study end points may have been impacted by the variability in the timing of when outcome assessments were collected. To reduce the burden on the study participants from having to complete the same assessments repeatedly in a short period of time, the study team made the decision to use recently collected data to serve as study end points when available and collected in close proximity to study activities. Although some assessments were then not collected immediately prior to starting the study intervention, the authors felt the period of time between admission assessments, study enrollment, and study initiation was sufficiently short enough, and completed consistently enough, to be representative and with minimal variation.

### Conclusions

PBT therapy is an important treatment modality for the rehabilitation of gait and balance following a stroke. As it becomes more accessible and widely adopted, ceiling-track-mounted BWSS with an integrated PBT module allows therapists to more safely add PBT into patients’ rehabilitation sooner and in ways that are more representative of their functional environment. Our goal was to determine if neurological rehabilitation paired with this technology could significantly improve poststroke gait, balance, confidence, and functional outcomes compared to using a BWSS alone. Ultimately, both were found to be similarly effective interventions, resulting in similar improvements in patient gait, balance, confidence, and functional outcomes. Given the safety it provides to clinicians and patients alike, conducting PBT with such a device provides a safe option for conducting gait and PBT in the inpatient setting, especially for patients who are unable to safely ambulate without assistance.

## Supplementary material

10.2196/80525 Checklist 1 CONSORT checklist.

## Abbreviations

10MWT: 10 Meter Walk Test
ABC: Activities-Specific Balance Confidence Scale
BBS: Berg Balance Scale
BWSS: body weight support system
BWSS-P: BWSS plus an integrated perturbation-based balance training module
PBT: perturbation-based balance training

## References

[1] (2023). Stroke facts. Centers for Disease Control and Prevention.

[2] Stroke information page. National Institute of Neurological Disorders and Stroke.

[3] Benjamin EJ, Blaha MJ, Chiuve SE (2017). Heart Disease and Stroke Statistics—2017 Update: a report from the American Heart Association. Circulation.

[4] Algurén B, Lundgren-Nilsson A, Sunnerhagen KS (2010). Functioning of stroke survivors—a validation of the ICF core set for stroke in Sweden. Disabil Rehabil.

[5] Chen N, Xiao X, Hu H, Chen Y, Song R, Li L (2019). Identify the alteration of balance control and risk of falling in stroke survivors during obstacle crossing based on kinematic analysis. Front Neurol.

[6] Forster A, Young J (1995). Incidence and consequences of falls due to stroke: a systematic inquiry. BMJ.

[7] Legters K (2002). Fear of falling. Phys Ther.

[8] Liphart J, Gallichio J, Tilson JK, Pei Q, Wu SS, Duncan PW (2016). Concordance and discordance between measured and perceived balance and the effect on gait speed and falls following stroke. Clin Rehabil.

[9] Asai T, Oshima K, Fukumoto Y, Yonezawa Y, Matsuo A, Misu S (2022). The association between fear of falling and occurrence of falls: a one-year cohort study. BMC Geriatr.

[10] Lee SW, Elsakr C, Ayutyanont N, Lee S, Oh-Park M (2023). Clinical characteristics and outcomes of inpatient falls during inpatient rehabilitation: a case-control study. Am J Phys Med Rehabil.

[11] Dykes PC, Curtin-Bowen M, Lipsitz S (2023). Cost of inpatient falls and cost-benefit analysis of implementation of an evidence-based fall prevention program. JAMA Health Forum.

[12] Hidler J, Hamm LF, Lichy A, Groah SL (2008). Automating activity-based interventions: the role of robotics. J Rehabil Res Dev.

[13] Meyer A, Hrdlicka HC, Cutler E (2022). A novel body weight–supported postural perturbation module for gait and balance rehabilitation after stroke: preliminary evaluation study. JMIR Rehabil Assist Technol.

[14] Choi W (2022). Effects of robot-assisted gait training with body weight support on gait and balance in stroke patients. Int J Environ Res Public Health.

[15] Anggelis E, Powell ES, Westgate PM, Glueck AC, Sawaki L (2019). Impact of motor therapy with dynamic body-weight support on functional independence measures in traumatic brain injury: an exploratory study. NeuroRehabilitation.

[16] Chien JE, Hsu WL (2018). Effects of dynamic perturbation-based training on balance control of community-dwelling older adults. Sci Rep.

[17] Esmaeili V, Juneau A, Dyer JO (2020). Intense and unpredictable perturbations during gait training improve dynamic balance abilities in chronic hemiparetic individuals: a randomized controlled pilot trial. J Neuroeng Rehabil.

[18] Steib S, Klamroth S, Gaßner H (2017). Perturbation during treadmill training improves dynamic balance and gait in Parkinson’s disease: a single-blind randomized controlled pilot trial. Neurorehabil Neural Repair.

[19] Schinkel-Ivy A, Huntley AH, Aqui A, Mansfield A (2019). Does perturbation-based balance training improve control of reactive stepping in individuals with chronic stroke?. J Stroke Cerebrovasc Dis.

[20] Mansfield A, Aqui A, Danells CJ (2018). Does perturbation-based balance training prevent falls among individuals with chronic stroke? A randomised controlled trial. BMJ Open.

[21] Shimada H, Obuchi S, Furuna T, Suzuki T (2004). New intervention program for preventing falls among frail elderly people: the effects of perturbed walking exercise using a bilateral separated treadmill. Am J Phys Med Rehabil.

[22] McCrum C, Bhatt TS, Gerards MHG (2022). Perturbation-based balance training: principles, mechanisms and implementation in clinical practice. Front Sports Act Living.

[23] Gerards MHG, McCrum C, Mansfield A, Meijer K (2017). Perturbation-based balance training for falls reduction among older adults: current evidence and implications for clinical practice. Geriatr Gerontol Int.

[24] Gerards MHG, Marcellis RGJ, Poeze M, Lenssen AF, Meijer K, de Bie RA (2021). Perturbation-based balance training to improve balance control and reduce falls in older adults - study protocol for a randomized controlled trial. BMC Geriatr.

[25] Hidler J, Lum PS (2011). The road ahead for rehabilitation robotics. J Rehabil Res Dev.

[26] Hidler J, Brennan D, Black I, Nichols D, Brady K, Nef T (2011). ZeroG: overground gait and balance training system. J Rehabil Res Dev.

[27] Gaylord Hospital, Inc (2023). A multisite exploration of balance perturbations with and without body weight support. NCT05110300.

[28] (2020). Berg Balance Scale. Shirley Ryan AbilityLab.

[29] (2013). Activities-Specific Balance cConfidence cale. Shirley Ryan AbilityLab.

[30] (2014). 10 Meter Walk Test. Shirley Ryan AbilityLab.

[31] Kyrdalen IL, Thingstad P, Sandvik L, Ormstad H (2019). Associations between gait speed and well-known fall risk factors among community-dwelling older adults. Physiother Res Int.

[32] Fox J, Weisberg S (2019). An R Companion to Applied Regression.

[33] Lenth RV, Banfai B, Bolker B Emmeans: estimated marginal means, aka least-squares means. R Project.

[34] Bates D, Mächler M, Bolker B, Walker S (2015). Fitting linear mixed-effects models using lme4. J Stat Softw.

[35] 9.5 - frequentist methods: o’brien-fleming, pocock, haybittle-peto. Eberly College of Science, Pennsylvania State University.

[36] Stevenson TJ (2001). Detecting change in patients with stroke using the Berg Balance Scale. Aust J Physiother.

[37] Dobkin BH (2004). Strategies for stroke rehabilitation. Lancet Neurol.

[38] Chayasit P, Hollands K, Hollands M, Boonsinsukh R (2022). Immediate effect of voluntary-induced stepping response training on protective stepping in persons with chronic stroke: a randomized controlled trial. Disabil Rehabil.

[39] Dusane S, Bhatt T (2022). Can prior exposure to repeated non-paretic slips improve reactive responses on novel paretic slips among people with chronic stroke?. Exp Brain Res.

[40] Dusane S, Wang E, Bhatt T (2019). Transfer of reactive balance adaptation from stance-slip perturbation to stance-trip perturbation in chronic stroke survivors. Restor Neurol Neurosci.

[41] Handelzalts S, Kenner-Furman M, Gray G, Soroker N, Shani G, Melzer I (2019). Effects of perturbation-based balance training in subacute persons with stroke: a randomized controlled trial. Neurorehabil Neural Repair.

[42] Marigold DS, Eng JJ, Dawson AS, Inglis JT, Harris JE, Gylfadóttir S (2005). Exercise leads to faster postural reflexes, improved balance and mobility, and fewer falls in older persons with chronic stroke. J Am Geriatr Soc.

[43] Mansfield A, Schinkel-Ivy A, Danells CJ (2017). Does perturbation training prevent falls after discharge from stroke rehabilitation? A prospective cohort study with historical control. J Stroke Cerebrovasc Dis.

[44] Brown D, Simpkins C, Yang F (2023). A systematic review of perturbation-based balance training on reducing fall risk among individuals with stroke. Clin Biomech (Bristol, Avon).

[45] (2020). Dynamic Gait Index. Shirley Ryan AbilityLab.

[46] (2016). Functional Gait Assessment. Shirley Ryan AbilityLab.

